# Plastome of the mycoheterotrophic eudicot *Exacum paucisquama* (Gentianaceae) exhibits extensive gene loss and a highly expanded inverted repeat region

**DOI:** 10.7717/peerj.9157

**Published:** 2020-06-09

**Authors:** Zhanghai Li, Xiao Ma, Yi Wen, Sisi Chen, Yan Jiang, Xiaohua Jin

**Affiliations:** 1 State Key Laboratory of Systematic and Evolutionary Botany, Institute of Botany, Chinese Academy of Sciences, Beijing, China; 2 University of Chinese Academy of Sciences, Beijing, China; 3 Nanchang University, Nanchang, China; 4 Southeast Asia Biodiversity Research Institute, Chinese Academy of Sciences (CAS-SEABRI), Xishuangbanna, China

**Keywords:** Mycoheterotrophy, *Exacum paucisquama*, Gentianaceae, Plastid genome, Gene loss, Inverted repeat region expansion

## Abstract

Mycoheterotrophic plants are highly specialized species able to acquire organic carbon from symbiotic fungi, with relaxed dependence on photosynthesis for carbon fixation. The relaxation of the functional constraint of photosynthesis and thereby the relaxed selective pressure on functional photosynthetic genes usually lead to substantial gene loss and a highly degraded plastid genome in heterotrophs. In this study, we sequenced and analyzed the plastome of the eudicot *Exacum paucisquama*, providing the first plastid genome of a mycoheterotroph in the family Gentianaceae to date. The *E. paucisquama* plastome was 44,028 bp in length, which is much smaller than the plastomes of autotrophic eudicots. Although the *E. paucisquama* plastome had a quadripartite structure, a distinct boundary shift was observed in comparison with the plastomes of other eudicots. We detected extensive gene loss and only 21 putative functional genes (15 protein-coding genes, four rRNA genes and two tRNA genes). Our results provide valuable information for comparative evolutionary analyses of plastomes of heterotrophic species belonging to different phylogenetic groups.

## Introduction

The characteristic feature of green plants is the presence of chloroplasts, which convert inorganic carbon to organic carbon by photosynthesis. However, a small number of plants have evolved a more specific type of nutrient acquisition strategy, namely heterotrophy. Heterotrophic plants can be subdivided into parasitic and mycoheterotrophic plants which acquire organic carbon from plant hosts or symbiotic fungi, respectively (Merckx et al., 2013a). Heterotrophy has evolved independently in plants numerous times, enabling the investigation of the loss of photosynthetic capability on a broad phylogenetic scale (Barrett et al., 2014; Barrett, Wicke & Sass, 2018; Logacheva et al., 2014; Feng et al., 2016; Lim et al., 2016; Wicke et al., 2016; Barrett & Kennedy, 2018; Petersen et al., 2018).

The relaxation of the functional constraint of photosynthesis results in the relaxed selective pressure on functional photosynthetic genes, which leads to the substantial gene loss and a highly degraded plastid genome in heterotrophs (Yuan et al., 2018). However, the reduction of the plastome size, gene loss and mutations are observed to varying degrees in heterotrophs (Delannoy et al., 2011; Logacheva et al., 2014; Molina et al., 2014; Lam, Soto Gomez & Graham, 2015; Schelkunov et al., 2015; Feng et al., 2016; Lim et al., 2016; Naumann et al., 2016; Graham, Lam & Merckx, 2017; Barrett & Kennedy, 2018; Petersen et al., 2018; Kim et al., 2019). General trends in gene loss observed in independently evolved heterotrophic lineages have provided a basis for the concept of a conserved core gene set, originally thought to include rRNA genes, ribosomal protein genes, several tRNA genes and four protein-coding genes (*clp*P, *acc*D, *ycf*1 and *ycf*2) (Delannoy et al., 2011; Logacheva, Schelkunov & Penin, 2011). Subsequently, Barrett & Davis (2012) proposed a model to describe the pattern of plastid gene loss in heterotrophs during plastome degradation (Barrett & Davis, 2012; Barrett et al., 2014). Based on recent studies, Graham, Lam & Merckx (2017) proposed a modified model, with broader windows for the retention of PEP, Rubisco and ATP synthase. Hence, plastid genome sequences of additional heterotrophic species from different phylogenetic clades are important to evaluate different scenarios of core gene retention in fully heterotrophic taxa.

The family Gentianaceae (order Gentianales) includes approximately 99 genera and 1,736 species. Of these, 25 species are putative full mycoheterotrophs, with at least four independent origins in *Voyria*, *Voyriella*, *Exacum* (including the formerly recognized genus *Cotylanthera*) and *Exochaenium* (Merckx et al., 2013a; Struwe, 2014). The mycoheterotrophic lifestyle has only been reported in Gentianaceae in the order Gentianales (Merckx et al., 2013b). However, plastid genomes of heterotrophic species in this family have not yet been characterized. Little is known about the modification of the plastid genome in the mycoheterotrophic Gentianales.

**Figure 1:**
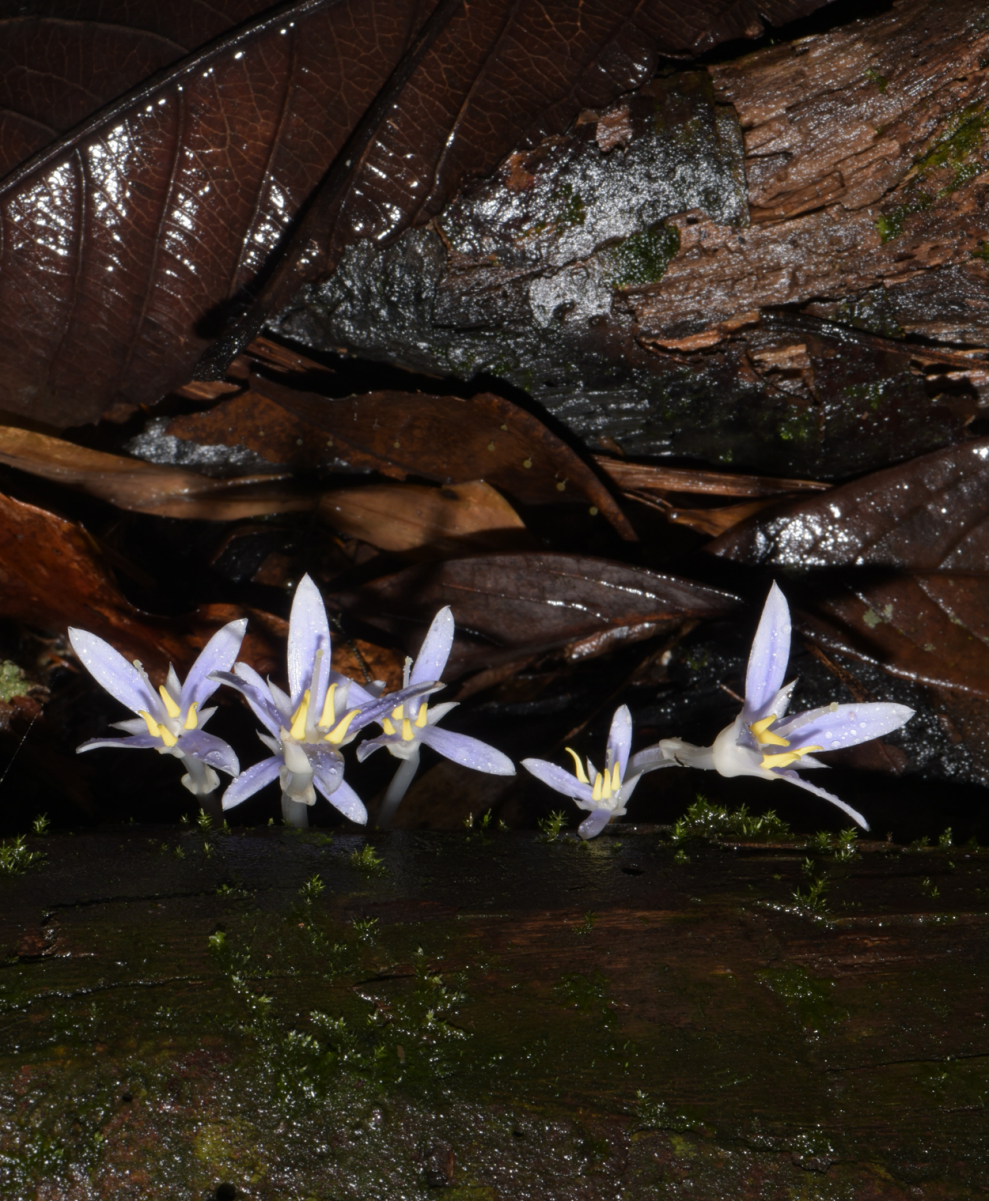
Image of the tiny, achlorophyllous eudicot *Exacum paucisquama* in its natural habitat. Photo: Xiao-Hua Jin.

In this study, we sequenced the complete plastome of *Exacum paucisquama* (Fig. 1), an achlorophyllous Gentianaceae species that parasitizes fungi to obtain nutrients (Leake, 1994; Merckx & Freudenstein, 2010). Our aims were to (1) explore the characteristics of the plastid genome of *E*. *paucisquama* and (2) compare the plastome of *E*. *paucisquama* with the plastomes of other heterotrophic plant lineages that have undergone independent losses of photosynthesis-related genes. The results of these analyses provide a more comprehensive understanding of plastome evolution in heterotrophic plants.

## Materials and Methods

Fresh stems and flowers were harvested from *E. paucisquama* plants growing in a field in Yunnan, China. Samples were dried in silica gel and preserved at −20 °C. Total DNA was isolated using a modified CTAB protocol (Li et al., 2013). DNA (>100 ng/ml) was sheared to fragments of 400–600 bp using Covaris M220. The NEBNext Ultra DNA Library Prep Kit was used to prepare DNA libraries for subsequent sequencing, according to the manufacturer’s protocol. Paired-end sequencing with a read length of 150 bp was performed using the Illumina HiSeq 2500 platform at the Institute of Botany, Chinese Academy of Sciences.

To clean the raw sequence reads, quality control was performed following the methods of Li et al. (2019). Plastomes were assembled from clean reads according to the methods of Feng et al. (2016) and Li et al. (2019). In short, clean reads were mapped to the plastome of *Gentiana straminea* (GenBank, KJ657732) using Geneious v10.2.2 (http://www.geneious.com, last accessed 4 May 2019) to filter reads matching the reference genome (Kearse et al., 2012). De novo assemblies were constructed using VELVET with several *K*-mer values (Zerbino & Birney, 2008), and contigs from each assembly were merged and combined into scaffolds in Geneious. Then, using the scaffolds as references to filter the plastome reads from clean reads, the assembly steps were repeated to obtain the draft plastome. Additionally, NOVOPlasty v.2.7.1 (Dierckxsens, Mardulyn & Smits, 2016), which uses a reference sequence (in this study, *acc*D from the leafy, photosynthetic gentian *G. straminea*) as an initial seed, was used to build a draft plastome. Reads were mapped with high stringency to the draft plastomes, produced by both assemblies, using Geneious to verify assembly errors. The plastome was first annotated using Geneious and GeSeq (Tillich et al., 2017), and tRNA genes were further predicted using tRNAscan-SE (Lowe & Chan, 2016). Start and stop codons, exon and intron boundaries and putative non-functional pseudogenes were identified and adjusted by aligning the plastome to protein-coding, tRNA and rRNA gene sequences of *G. straminea*. The circular plastome map was visualized using OGDRAW v1.2 (Lohse et al., 2013).

The plastome of *E. paucisquama* was aligned with the plastomes of three species, *G. straminea*, *Halenia corniculata* (GenBank, MK606372), and *Swertia verticillifolia* (GenBank, MF795137), using the progressiveMAUVE (Darling, Mau & Perna, 2010) plugin for Geneious to identify syntenic blocks and thereby to detect genomic rearrangements. Putative functional genes (i.e., genes with open reading frames), pseudogenes (i.e., genes with interrupted open reading frames or nontriplet nucleotide indels), and physical gene losses were identified by comparisons to the plastome of the leafy, photosynthetic relative *G. straminea*. To compare the plastome size and functional gene content among fully heterotrophic species, plastome sequences of 17 heterotrophs were downloaded from the NCBI database (Table S1).

## Results and Discussion

**Figure 2:**
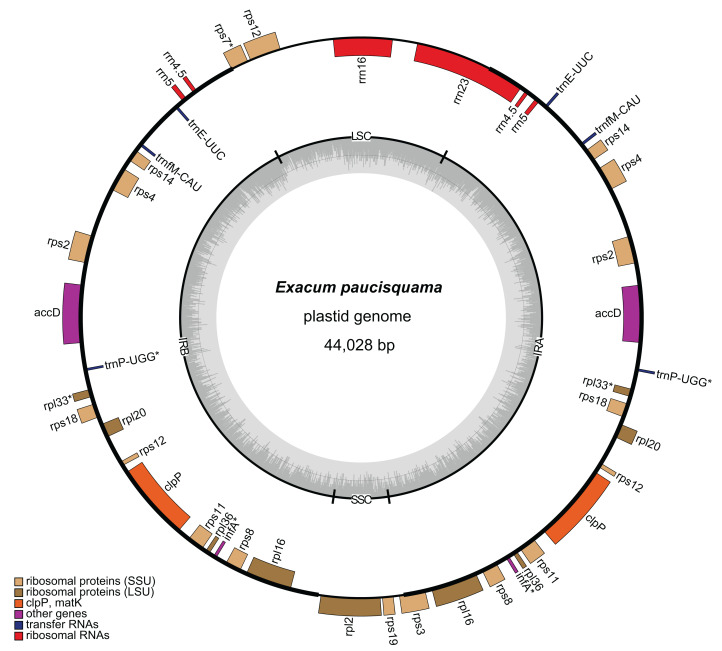
Circular map of the plastome of *E. paucisquama*. Asterisks (*) indicate pseudogenes. Thick lines indicate the extent of the inverted repeat regions (IRa and IRb), which separate the genome into small (SSC) and large (LSC) single copy regions. Genes drawn inside the circle are transcribed clockwise, while those outside of the circle are transcribed counter clockwise. Genes belonging to different functional groups are color coded. Dark gray in the inner circle corresponds to the GC content, while light gray corresponds to the AT content.

A total of 6,455,059,021 clean reads, with an average length of 150 bp, were recovered from 7,147,180,924 raw reads. Among the clean reads, 32,399,763 (0.50%) corresponded to the plastome. The average coverage depths were 644× and 1,359× in single-copy (SC) regions and inverted repeat (IR) regions, respectively (Fig. S1). The assembled plastome of *E. paucisquama* has been deposited in NCBI GenBank under accession number MN067514. The complete plastome of *E. paucisquama* was 44,028 bp in length, with a quadripartite structure (Fig. 2). It had a basically high degree of collinearity with the genome of its autotrophic relatives (Fig. S2). The plastid genome of *E. paucisquama* contained 21 putative functional genes, including 13 ribosomal protein genes, *clp*P, *acc*D. four rRNA genes and two tRNA genes (*trn*E and *trn*fM). The total GC content of the *E. paucisquama* plastome was 37.1%, after removing one copy of the IR region (Table S1).

The IR region is hypothesized to stabilize the plastome (Palmer & Thompson, 1982) and is retained in most sequenced plastomes, including those of heterotrophic species with extensive gene loss (Lim et al., 2016; Schelkunov et al., 2015). Unlike the plastomes of autotrophs in Gentianaceae, more than 80% of the plastome of *E. paucisquama* was classified as IRs (2 × 17,622 bp), and the IR regions harbored most of the genes, starting with *rps*3 and ending with a part of *rrn*23/*rps*7 (Fig. 2). IRs have a major impact on the rate of plastome sequence evolution; in general, the rate of nucleotide substitution is several times lower in IR than in SC regions (Zhu et al., 2016), and genes translocated from the SC into the IR region show decelerated substitution rates in the fern (Li et al., 2016).

The large single copy (LSC) region of the *E. paucisquama* plastome had a length of 6,651 bp and contained *rrn*16, the 3′ end of *rps*12, a part of *rrn*23, and a part of *rps*7. The small single copy (SSC) region was 2,133 bp in length and contained only *rpl*2, *rps*19, and a part of *rps*3. Generally, in plastid genomes, each IR region contains three to five tRNA genes, four *rrn* genes, two *rps* genes and *ycf*2, and the SSC region contains six *ndh* genes, *rpl*32, *psa*C, *rps*15, *ycf*1 and *trn*L^UAG^. However, the *E. paucisquama* plastome showed a shift in the boundaries between the IR and SC regions. All of the genes usually present in the SSC region were translocated to the IR region, and some of the sequences usually contained in IR regions were located in the SC regions. The boundary shift led to the presence of a single copy of *rrn*16 and partial *rrn*23, in contrast to the duplicates in IR regions observed in most plastomes.

The extensive loss of plastid genes in *E. paucisquama* corresponds to the final stage of the plastome degradation model proposed by Barrett & Davis (2012). Some “housekeeping” genes, such as *ycf*1, *ycf*2, *mat*K and many tRNA genes, present in most mycoheterotrophic species, were lost in the *E. paucisquama* plastome. The *E. paucisquama* plastome contains only two tRNA genes: *trn*fM^CAU^ and *trn*E^UUC^_._ The loss of some tRNA genes may be compensated for by the import of tRNAs from the cytosol (Alkatib et al., 2012) or by ‘superwobbling’ (Rogalski, Karcher & Bock, 2008). The gene product of *trn*E^UUC^ (glutamyl-tRNA) plays a secondary role in heme biosynthesis (Jahn, Verkamp & So, 1992) and may regulate the translation of the nuclear-encoded plastid RNA synthase (NEP) (Hanaoka et al., 2005). Barbrook, Howe & Purton (2006) proposed that the interaction of *trn*E^UUC^ with multiple enzymes involved in heme biosynthesis makes its replacement by a cytosolic product unlikely. *trn*fM^CAU^, plastid-encoded formylmethionyl-tRNA, regulates the translation initiation in plastids and possibly in mitochondria (Barbrook, Howe & Purton, 2006). The indispensable nature of these two plastid-encoded tRNAs could explain the retention of plastomes in non-photosynthetic organisms; this hypothesis is referred to as the essential tRNA hypothesis (Barbrook, Howe & Purton, 2006). The *mat*K gene encodes the only plastid-encoded group IIa intron maturase, MATK (Zoschke et al., 2010). Although *mat*K is present in nearly all plant plastid genomes, it has been deleted from the plastome of *E. paucisquama*. However, the *E. paucisquama* plastome retained loci with group IIa introns, and at least two of these genes (*rpl*2 and *rps*12) are thought to be targeted by MATK (Zoschke et al., 2010). It is possible that an alternative splicing factor facilitates intron removal from their RNA transcripts, as proposed by Delannoy et al. (2011), based on the observation that *rpl*2 was correctly spliced in the case of a *mat*K gene deletion in the *Rhizanthella gardneri* plastome.

**Figure 3:**
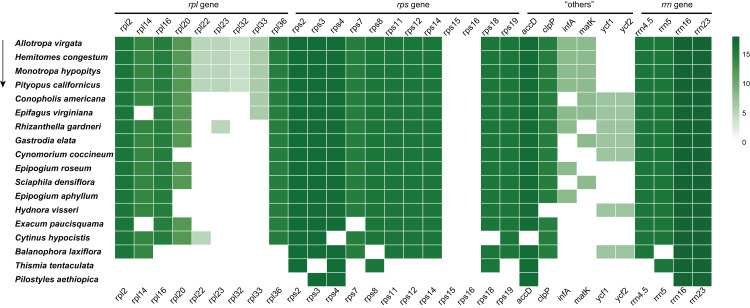
Summary of the *rpl*, *rps*, “others” (*acc*D, *clp*P, *inf*A, *mat*K, *ycf*1 and *ycf*2), and *rrn* genes in the plastome of *E. paucisquama* and 17 other fully heterotrophic species. The arrow indicates the degree of gene loss. Green boxes represent the retained putatively functional genes; a deeper green color indicates a higher number of species retaining the genes.

In the *E. paucisquama* plastome, only two protein-coding genes, *acc*D and *clp*P. involved in functions other than translation, were retained, similar to other non-photosynthetic plant species with a highly reduced plastome (Fig. 3), such as *Epipogium* (Schelkunov et al., 2015). The *acc*D gene plays an important role in fatty acid biosynthesis (Bryant et al., 2011), and the loss of *acc*D from the plastome of heterotrophic plants is a rare event (Lim et al., 2016). The *clp*P gene encodes a subunit of the Clp protease (or ATP-dependent caseinolytic protease), involved in the regulation of protein turnover and processing and has also been linked to isoprenoid and tetrapyrrole biosynthesis and fibrillin (lipid-body stabilizing molecules) (Kim et al., 2009; Stanne et al., 2009). Notably, both of these genes participate in the regulation of lipid metabolism, supporting the role of lipids in sustaining colonization by mutualistic mycorrhizal and parasitic fungi (Jiang et al., 2017). In the *E. paucisquama* plastome, these two genes were duplicated along with the expansion of the IR region. Expanded IR has been observed in other mycoheterotrophs (Naumann et al., 2016; Joyce et al., 2019) and autotrophs (Zhu et al., 2016; Sinn et al., 2018), which leads to a decelerated substitution rates for the genes translocated from the SC into the IR region in fern (Li et al., 2016). Biased gene conversion, which means new mutations corrected back to ancestral states preferentially (Birky & Walsh, 1992), is hypothesized as the reason behind the lower rate of nucleotide substitution in the IR region (Wu & Chaw, 2015; Li et al., 2016; Zhu et al., 2016). The expansion of the IR region in *E. paucisquama* may be a mechanism for the prevention of immoderate mutations to retain the functionality of genes translocated to the IR region. The movement of genes in SC region to the IR region could be an advantageous move that is, selected for in *E. paucisquama*. This coincides with the view that the degradation of plastomes in mycoheterotrophic species presents a highly lineage-specific pattern (Feng et al., 2016).

## Conclusion

We report the first plastid genome of mycoheterotrophic species in the family Gentianaceae sequenced to date. The *E. paucisquama* plastome showed extensive gene losses and contained only 21 putative functional genes (15 protein-coding genes, four rRNA genes and two tRNA genes). Some “housekeeping” genes, such as *ycf*1, *ycf*2, *mat*K and many tRNA genes, were lost in the *E. paucisquama* plastome. More than 80% of the plastome of *E. paucisquama* is IR regions, and these regions harbor most of the remaining genes. Our results provide valuable information for the comparative evolutionary analyses of plastomes of heterotrophic species belonging to different phylogenetic lineages.

## Supplemental Information

10.7717/peerj.9157/supp-1 Supplemental Information 1 Coverage of sequencing data mapped to the assembled plastome of *Exacum paucisquama*.

10.7717/peerj.9157/supp-2 Supplemental Information 2 Alignment of the plastomes of *Gentiana straminea* (KJ657732), *Halenia corniculata* (MK606372), *Swertia verticillifolia* (MF795137), and *E. paucisquama* (MN067514) using progressive MAUVE.

10.7717/peerj.9157/supp-3 Supplemental Information 3 Length and GC content of the newly sequenced plastome and 17 publicly available plastomes.

10.7717/peerj.9157/supp-4 Supplemental Information 4 GO annotations.

10.7717/peerj.9157/supp-5 Supplemental Information 5 The complete plastid genome of Exacum paucisquama.

## Additional Information and Declarations

### Data Availability

The following information was supplied regarding data availability: The complete plastid genome sequence of Exacum paucisquama is available as a Supplemental File and at GenBank: MN067514.
