# Supplementary figures and images for: Plastome of the mycoheterotrophic eudicot Exacum paucisquama (Gentianaceae) exhibits extensive gene loss and a highly expanded inverted repeat region
Source: PeerJ. 2020 Jun 9;8:e9157. doi: 10.7717/peerj.9157 (PMC7292021; doi:10.7717/peerj.9157)

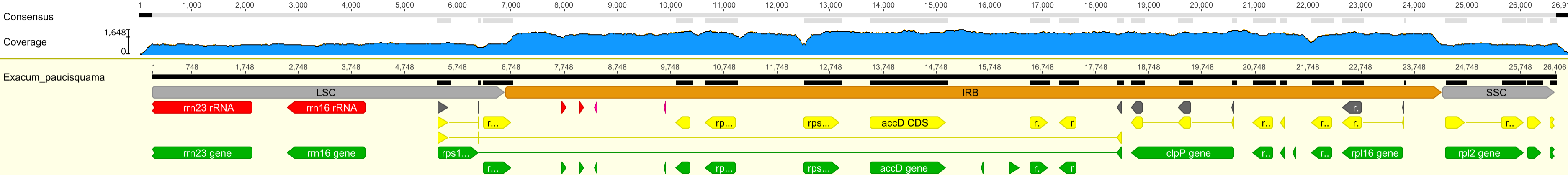

Supplement: Supplemental Information 1 [file peerj-08-9157-s001.pdf]

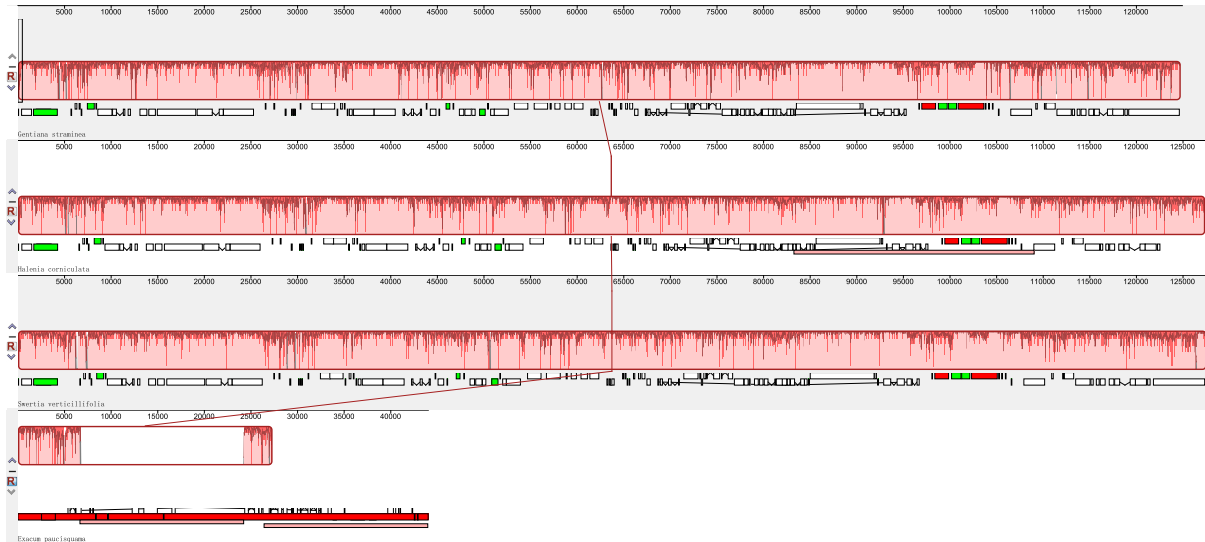

Supplement: Supplemental Information 2 [file peerj-08-9157-s002.pdf]
